# EPIXplorer: A web server for prediction, analysis and visualization of enhancer-promoter interactions

**DOI:** 10.1093/nar/gkac397

**Published:** 2022-05-25

**Authors:** Li Tang, Zhizhou Zhong, Yisheng Lin, Yifei Yang, Jun Wang, James F Martin, Min Li

**Affiliations:** Hunan Provincial Key Lab on Bioinformatics, School of Computer Science and Engineering, Central South University, Changsha 410083, China; Hunan Provincial Key Lab on Bioinformatics, School of Computer Science and Engineering, Central South University, Changsha 410083, China; Hunan Provincial Key Lab on Bioinformatics, School of Computer Science and Engineering, Central South University, Changsha 410083, China; Hunan Provincial Key Lab on Bioinformatics, School of Computer Science and Engineering, Central South University, Changsha 410083, China; Department of Pediatrics, McGovern Medical School, The University of Texas Health Science Center at Houston, Houston, TX 77030, USA; Department of Molecular Physiology and Biophysics, Baylor College of Medicine, Houston, TX 77030, USA; Cardiovascular Research Institute, Baylor College of Medicine, Houston, TX 77030, USA; Texas Heart Institute, Houston, TX 77030, USA; Hunan Provincial Key Lab on Bioinformatics, School of Computer Science and Engineering, Central South University, Changsha 410083, China

## Abstract

Long distance enhancers can physically interact with promoters to regulate gene expression through formation of enhancer-promoter (E-P) interactions. Identification of E-P interactions is also important for profound understanding of normal developmental and disease-associated risk variants. Although the state-of-art predictive computation methods facilitate the identification of E-P interactions to a certain extent, currently there is no efficient method that can meet various requirements of usage. Here we developed EPIXplorer, a user-friendly web server for efficient prediction, analysis and visualization of E-P interactions. EPIXplorer integrates 9 robust predictive algorithms, supports multiple types of 3D contact data and multi-omics data as input. The output from EPIXplorer is scored, fully annotated by regulatory elements and risk single-nucleotide polymorphisms (SNPs). In addition, the Visualization and Downstream module provide further functional analysis, all the output files and high-quality images are available for download. Together, EPIXplorer provides a user-friendly interface to predict the E-P interactions in an acceptable time, as well as understand how the genome-wide association study (GWAS) variants influence disease pathology by altering DNA looping between enhancers and the target gene promoters. EPIXplorer is available at https://www.csuligroup.com/EPIXplorer.

## Graphical Abstract

**Graphical Abstract:**
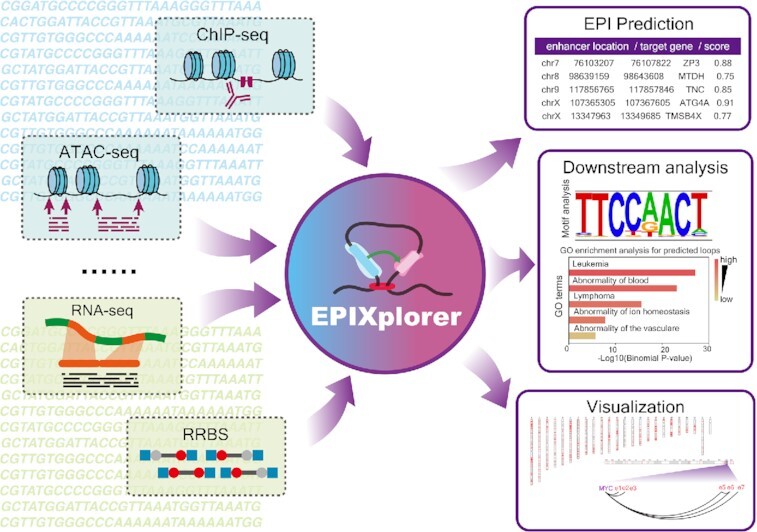
EPIXplorer is a user-friendly web server for efficient prediction, analysis and visualization of enhancer-promoter interactions. EPIXplorer integrates 9 robust predictive algorithms, supports multiple types of 3D contact data and multi-omics data as input. The output from EPIXplorer is scored, fully annotated by regulatory elements and risk single-nucleotide polymorphisms (SNPs). The Visualization and Downstream module provide further functional analysis.

## INTRODUCTION

Enhancers play an important role in driving gene expression patterns in a cell type specific manner as well as morphological differences (1). Many enhancers regulate gene expression through E-P interactions from a long genomic distance (2). Moreover, most of identified disease-associated genetic variants, locate in non-coding intergenic regions that often lie within or proximal to enhancer elements. Identifying which promoters topologically engage disease-associated mutated loci can offer important insight into how these polymorphisms contribute to disease risk (3–5). Modern Chromosome Conformation Capture (3C)-based assays facilitate the identification of such long-range contacts between disease loci and target promoters, such as High-throughput Chromosome conformation capture (Hi-C) (6), Capture Hi-C (7), Chromatin Interactive Analysis by Paired-End Tag Sequencing (ChIA-PET) (8), HiChIP (9), etc. However, these assays are technically challenging, expensive, and time-consuming, making it a great challenge to explore the chromosome contacts of unrecognized cell line or species.

Recently, some predictive computation tools are developed to solve the wet-lab experimental difficulties (10). Two state-of-art strategies taken by current computational methods were unsupervised-learning and supervised-learning, the unsupervised learning methods used inherent genomic patterns to predict chromatin interactions, such as distance or correlations between regulatory elements (11–13). PreSTIGE (12) linked cell type–specific enhancers to their target genes via the linear domain model. Ernst et al. (14) and Thurman et al. (15) utilized the correlations between promoter DNaseI-hypersensitive sites (DHS) and enhancers or the expression levels in specific regulatory regions. EpiTensor (16) employed a decomposition-based model to identify the interaction between promoters and enhancers. The supervised learning methods usually took classical learning models, such as random forest, neural network, decision tree, logistic regression, which trained the model with sequence or epigenomic data in a specific cell line, then applied the model to a similar or another cell line (17,18). IM-PET (19) integrated multiple genomic features to distinguish E-P pairs with random forest model. JEME (20) identified the interactions with linear regression model, which used epigenomics and expression features. TargetFinder (21) was a popular method for predicting E-P interaction in a cell-type specific manner, which used open chromatin information, gene expression, transcription factors (TFs), and histone marks to train the classification model. 3DPredictor (22) provided quantitative prediction of chromatin interactions by using CTCF binding signals and gene expression. LoopPredictor (23) was an ensemble machine learning model, can be used to predict enhancer mediated interactions in a genome-wide fashion across different cell lines and organisms, which provided both classification and regression of chromatin interactions.

To a certain extent, these computational methods effectively solve the difficulties of identifying enhancer-mediated chromatin interactions, while single method cannot meet various requirements of usage (Table 1). In addition, limited by the numerous inputs, excessive memory usages and overlong training time, these methods were hard to be widely used. Therefore, a user-friendly web server is necessary to simplify the computation procedure and facilitate further exploration of E-P interactions. With this in mind, we develop EPIXplorer, a web server for prediction, downstream analysis and visualization of E-P interactions, which integrates 9 robust predictive algorithms. EPIXplorer supports multiple types of 3D contact data (Hi-C, ChIA-PET, HiChIP, etc), and multi-omics data (ChIP-seq, RRBS, ATAC-seq, RNA-seq, etc.) as input, and provides scoring, annotation for the predicted results with regulatory elements and risk SNPs. Additionally, the Visualization and Downstream module facilitate the functionality exploration of loops. Overall, EPIXplorer can predict the enhancer connectomes of uncharacterized cell types, explore the functional complexity of 3D genome, as well as investigate the pathological mechanism of GWAS variants in the context of 3D genome.

**Table 1.** The predictive algorithms integrated in EPIXplorer

	Strategy	Input	Prediction output	Downstream analysis/ Visualization	Advantage	Disadvantage
PreSTIGE	Unsupervised (Distance-based)	Distance, H3K4me1, RNA-seq	E-P interaction	No/No	Low running times, does not need 3D contact	Low accuracy
Ernst et al.	Unsupervised (Correlation-based)	CTCF, histone marks, TF binding	E-P interaction	No/No	Low running times, does not need 3D contact	Low accuracy
Thurman et al.	Unsupervised (Correlation-based)	DHS	E-P interaction	No/No	Low running times, does not need 3D contact	Low accuracy
EpiTensor	Unsupervised (Decomposition-based)	DHS, histone marks, RNA-seq	3D interactions	No/No	does not need 3D contact	Low accuracy, slow speed
IM-PET	Supervised (Random Forest)	DNA, histone marks, TFBSs, RNA-seq + ChIA-PET	E-P interaction	No/No	High accuracy	Need enhancer locus and signals, classification only
JEME	Supervised (Linear Regression)	DHS, distance, eRNA, histone marks + ChIA-PET/Hi-C/eQTL	E-P interaction	No/No	High accuracy, does not need 3D contact	Slow speed, classification only
TargetFinder	Supervised (Gradient Tree Boosting)	DHS, DNA methylation, TFBSs, histone marks, CAGE + Hi-C	E-P interaction	No/No	High accuracy	Need 3D contact, classification only
3DPredictor	Supervised (Gradient Boosting)	CTCF, distance, RNA-seq + Hi-C	3D interactions	No/No	High accuracy	Need 3D contact, slow speed, classification only
LoopPredictor	Supervised (Random Forest, Gradient Boosted Regression Trees)	RNA-seq, ChIP-seq, ATAC-seq, RRBS + HiChIP	E-P interaction, Enhancer-Enhancer (E-E) interaction, Promoter-Promoter (P-P) interaction	No/No	High accuracy, both classification and regression	Need 3D contact, slow speed

## RESULTS

### Overall design of EPIXplorer

**Figure 1.**
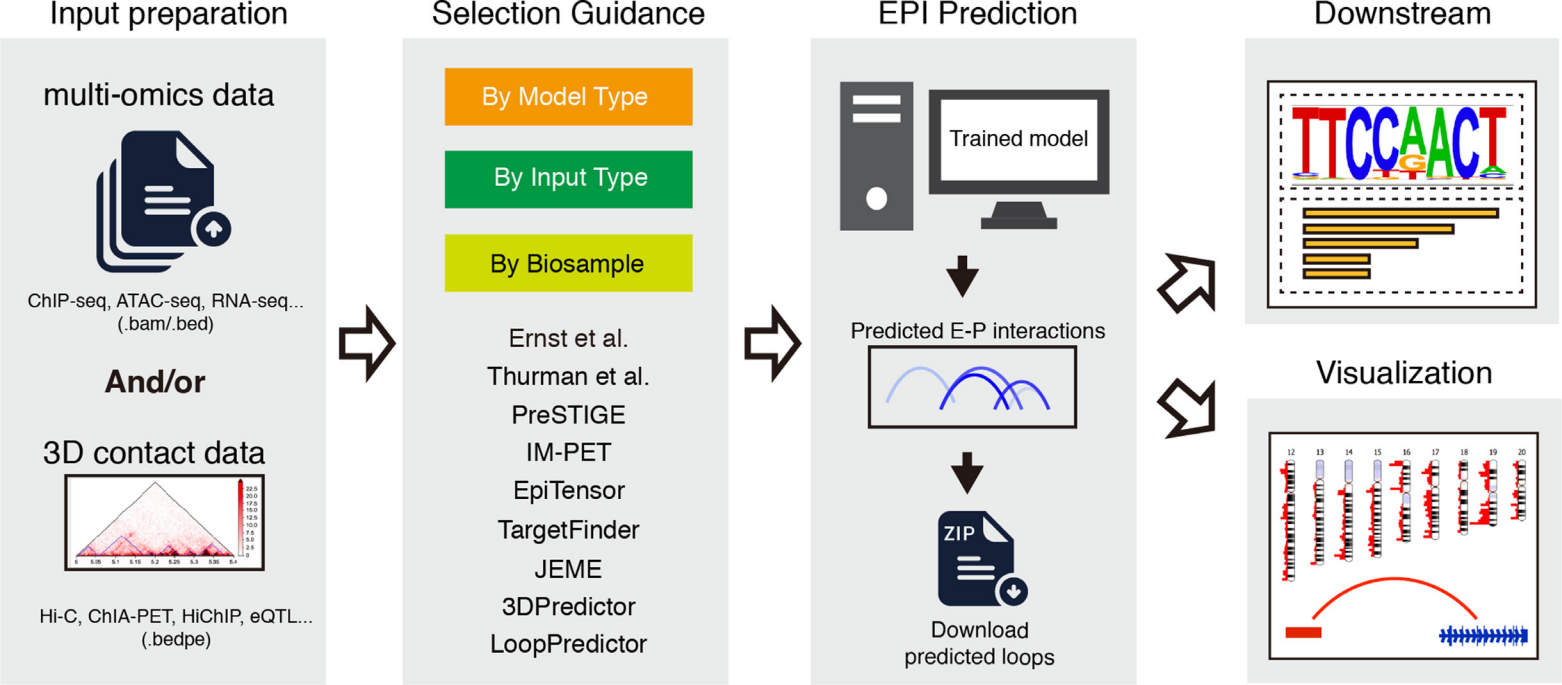
The overall design of EPIXplorer. Multi-omics datasets and/or 3D contact data can be prepared as input. EPIXplorer provides a practical guidance from three aspects: By Model Type (BMT), By Input Type (BIT), and By Bio Sample (BBS). Downstream and visualization modules perform further analysis for predicted loops.

The purpose of EPIXplorer is to facilitate the prediction of E-P chromatin interactions. The overall design of EPIXplorer is summarized in Figure 1. The web server accepts different types of input to meet the requirements of users, such as without any data input, with only multi-omics dataset, or with only 3D contact data. The multi-omics datasets include, but not limited to, RNA-seq gene expression profile, Transcription Factor ChIP-seq peaks, Histone Marks ChIP-seq peaks, and ATAC-seq peaks. The 3D contact data can be chromatin interactions captured by Hi-C, ChIA-PET, HiChIP, etc. Users can prepare the input files following the format description on tutorial page.

The web server integrates 9 robust algorithms to perform the prediction. To facilitate the selection of algorithms, we provide practical guidance from three aspects: By Model Type (BMT), By Input Type (BIT), and By Bio Sample (BBS) (Supplementary Figure S1). Following statements about the performance are explained later in the section ‘Performance of EPIXplorer’. BMT divides 9 algorithms into supervised and unsupervised according to the type of prediction model. The unsupervised models predict the connections between distal regulatory elements and genes based on the distance or the natural patterns of DNA sequences, which make the model easy to construct and save a lot of running time, while the accuracies of unsupervised models are relatively lower than the supervised models. For the unsupervised algorithms, we recommend PreSTIGE, which achieves relative higher accuracy than the other unsupervised models. For the supervised algorithms, the performance of LoopPredictor is the best. BIT characterizes these algorithms by the input they support. If users have both 3D contact and multi-omics data, LoopPredictor can be a good option, the prediction accuracy of which is generally higher than the others. If users only have epigenomic data, IM-PET can be chosen. If there is no input file prepared, the server provides ‘no upload’ mode to convenient the usage. In this mode, users can obtain the predicted loops by selecting a specific cell line and the interested gene/locus, without uploading any data. This mode was implemented by two ways, one is to integrate the predicted loops from publications (such as Ernst et al., Thurman et al.). The other is to collect the genomic distance and histone marks datasets of some common cell types, then perform the prediction in advance. Since the unsupervised methods don’t need 3D contact data as input, and no pre-training models required for the prediction, these methods are suitable implemented by the ‘no upload’ mode (such as EpiTensor, PreSTIGE). In addition, most of the algorithms can only be applied to the cell types in which they were trained, here BBS classifies all the supported cell lines into 9 major types and lists the available algorithms for each cell line. Next, the server executes the prediction with selected algorithm, the predicted E-P interactions are fully annotated and scored by confidence, which could be downloaded directly, or deposit to Downstream and Visualization module for further analysis.

Downstream module accepts both predicted E-P interactions and published 3D contact loops as input, this module provides GO analysis and motif analysis for users to explore the biological function of loops. In Visualization module, the genome-wide distribution of loops can be visualized by ideogram, the related regulatory elements and risk SNPs within interested locus are annotated. The analysis results from downstream and visualization module could be exported as high-quality images and available for download.

### Performance of EPIXplorer

**Figure 2.**
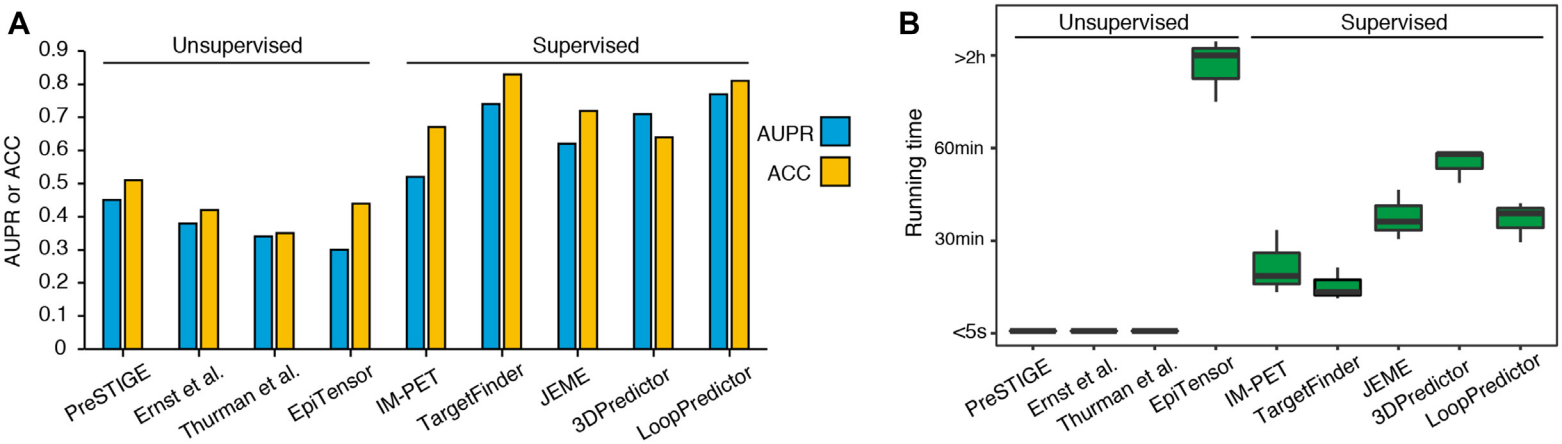
Performance of EPIXplorer. (**A**) The AUPR score and ACC of 9 integrated algorithms in EPIXplorer evaluated with K562 and GM12878 BENGI datasets and gold standard loop sets. (**B**) The running time of 9 integrated algorithms in EPIXplorer.

To evaluate the prediction performance of 9 integrated algorithms in EPIXplorer, two metrics were employed: the area under the precision recall curve (AUPR) and accuracy (ACC). In the study of Cao (24), AUPR is used to measure the performance of E-P interaction predictive methods, and AUPR is sensitive to unbalanced data in which instances are unequal for different classes (25). Here we employed K562 and GM12878 BENGI datasets (26) to calculate the AUPR score with 10-fold cross validation, the separation of positive and negative samples followed the description of Moore et al. (26) (Supplementary Table S1). For the supervised algorithms, four epigenomic features, including ChIP-seq (H3K4me1, H3K27ac and H3K27me3), DNase-seq/ATAC-seq data were applied. For the unsupervised algorithms, genomic distance or correlation were applied as required by different algorithms. In our previous work (27), the gold standard loops were collected from the GEUVADIS Project, GTEx Project, ENCODE project, and CRISPRi perturbation screening, which were regarded as the true loops, and a computational benchmark framework was proposed to evaluate the ChIA-PET/HiChIP data processing methods. Here we used the K562 and GM12878 cell lines of gold standard loops to evaluate EPIXplorer (Supplementary Table S2). Although the input and the strategies taken by the benchmarked methods were distinct from the predictive algorithms in our web server, the purposes were identical to obtain a set of accurate loops in specific cell type. Thus, we use the same accuracy metrics (ACC) and the gold standard datasets to measure the predicted loops. The evaluation results showed that the AUPR and ACC of supervised algorithms were generally higher than 0.6 (Figure 2A), which outperformed unsupervised algorithms, the results were consistent with the finding of Moore et al. (26).

To evaluate the running time of EPIXplorer, we repeated the prediction procedure with different number of inputs. For unsupervised algorithms, PreSTIGE, Ernst et al., and Thurman et al. were based on the calculation of distance or correlation, which weren’t impacted by the increasing of inputs, and took less than 5 seconds to finish. Since EpiTensor used a decomposition model, which took the most time to process the calculation. For supervised algorithms, all the prediction procedure can be finished within 1 hour (Figure 2B).

### Case study

EPIXplorer provided running case for each tool, and users can try the case by clicking ‘Load an example’ on the web interface. Here we ran the K562 datasets with LoopPredictor tool as an example since the prediction performance of LoopPredictor outperformed the other tools in this dataset (Supplementary Figure S2). To facilitate the interpretation of training and predicting procedure, the corresponding input data, generated features, and trained model were available for download. If users start a new prediction job by inputting their own data, the web server will assign a unique job id, and the prediction results will be stored in web server for a week, users can download or visualize the results through this job id.

**Figure 3.**
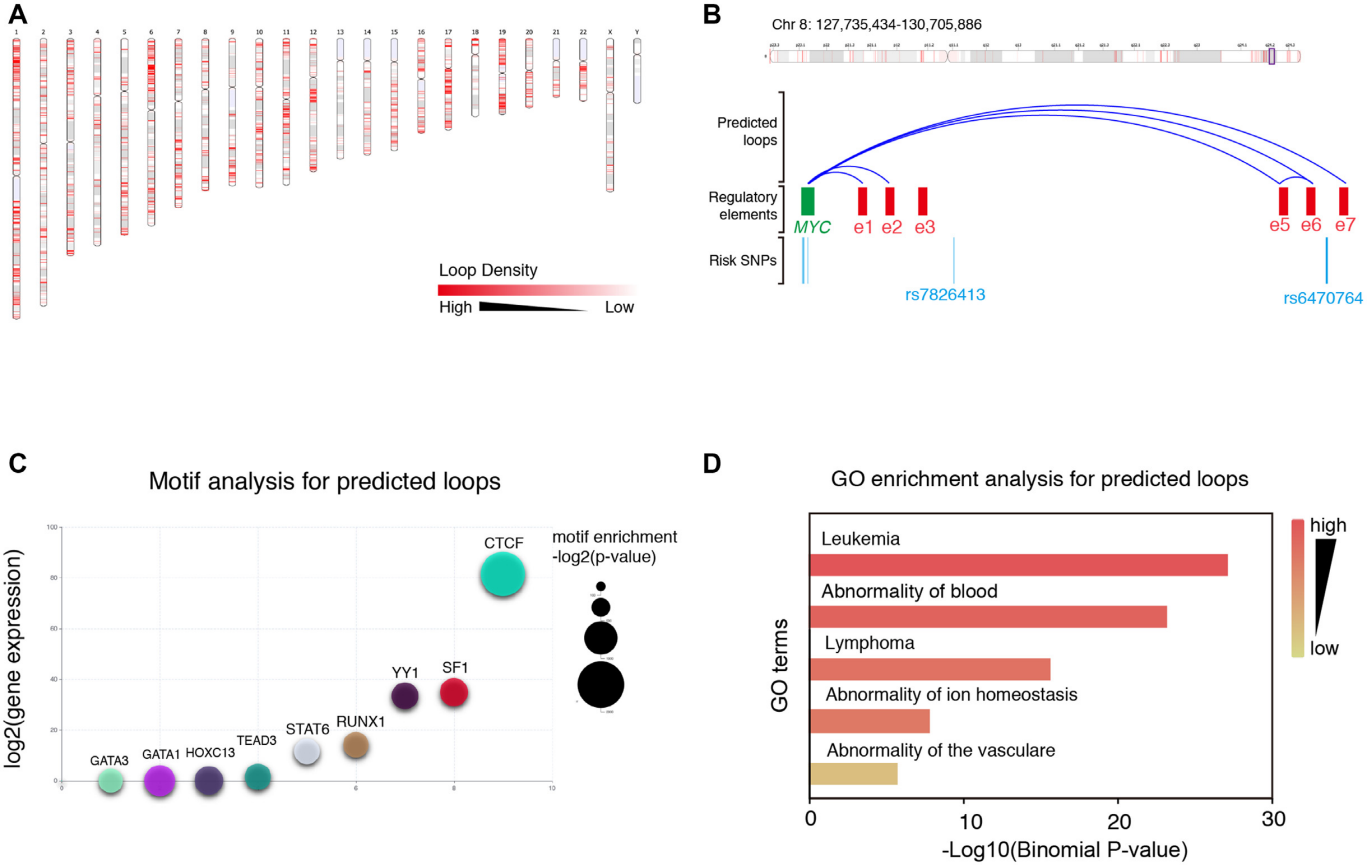
Example application of EPIXplorer implemented with LoopPredictor. (**A**) The genome-wide distribution of K562 predicted loops. (**B**) Validation of predicted loops by published CRISPRi contacts. (**C**) Ranking list of significantly enriched motifs. (**D**) Top enriched GO terms for predicted loop anchors.

When the running procedure finished, the predicted K562 loops could be visualized in genome-wide scale, in which the distribution of loops was showed with red lines on the genome bars, and the colour of lines indicated the density of loops (Figure 3A). In the study of Fulco (28), the interactions between *MYC* and 7 enhancers were identified via a systematic CRISPR interference (CRISPRi) screen. To check the consistence of predicted loops and verified CRISPRi contacts, we chose the region near *MYC* gene to visualize the loops, the enhancers were annotated as e1 through e7, which indicated the prediction results of EPIXplorer were in accordance with the published loops. The annotation results also indicated the elements predicted to regulate MYC harbored SNPs associated with human traits including Hodgkin's lymphoma (rs7826413) and height (rs6470764) (Figure 3B).

To explore the biological function of predicted loops, we performed downstream analysis based on the K562 predicted loops. Motif analysis was taken accompanying with RNA-seq expression data, the ranking list of significantly enriched motifs were showed with bubble plot, which indicated that the CTCF, SF1 and YY1 motifs were significantly enriched (-log2(p-value) < 100) and were highly expressed in K562 cells (Figure 3C). We then performed the GO enrichment analysis for the loop anchors, the enriched terms (such as leukemia, abnormality of blood) showed the loops contributed to the cell identity of K562 cell line, which demonstrated the effectiveness of EPIXplorer (Figure 3D). All the downstream analysis results visualized on the web interface were available for download.

## MATERIAL AND METHODS

### Implementation of the algorithms

We implemented all the algorithms with their original scripts/packages, which could be run normally on Ubuntu 18. To solve the environment conflicts between packages, we created an independent anaconda (https://docs.anaconda.com/) environment for each algorithm, the specific environment was loaded by homemade scripts. To make the server extendable and robust, we adopted a detachable architecture, which decomposed the server into front end and back end independently. The functional modular design ensured the operation of algorithms undisturbed and make it easy to integrate new function or algorithms.

### Generating features from multi-omics datasets

To gather the features into an integrated matrix for model training, the web server firstly collected multi-omics datasets from web interface, the format detection module was used to classify the input files into peaks (ChIP-seq, CUT&RUN, ATAC-seq, etc.) or expression profiles (RNA-seq). For the type of peaks, the informative columns of peak coordinates and peak scores were extracted and added into feature library. For the type of expression profiles, which included only gene names and corresponding expression values, we extracted the coordinates of genes by GENCODE v19 (29) annotation file, then added into feature library accordingly. Then we obtained the candidate promoter elements and enhancer elements from ENCODE (3) to build a regulatory library in the cell type specific manner. Next, we overlaid the feature library with regulatory library by bedtools (30) with the function of intersect, through which the feature vector of regulatory elements was generated. In the study of Whalen (21), the window regions between two anchors were more informative, and benefit to the prediction. Therefore, we extended each feature vector to the left-flanking, in-between, right-flanking regions for anchors, then all the feature vectors were merged into a feature matrix for the subsequent procedure.

### Training procedure

The feature matrix generated from regulatory elements were regarded as positive samples, and the annotations of regulatory elements for anchors were used as the target of samples, we only retained four types of targets for the prediction: enhancer-promoter (E-P), promoter-promoter (P-P), enhancer-enhancer (E-E), and none. The type of E-P indicated one of the two anchors was promoter, and the other was enhancer, P-P and E-E indicated both anchors were promoters or enhancers. The type of none-none represented the anchors of loop were uncertain type, including either of two anchors or both anchors are non-regulatory elements. Besides, negative regions were selected randomly by avoiding ± 2kb around TSS locus of any gene. Then the selected negative regions were used to overlay with the feature library as mentioned above. The amount of negative sample was consistent with positive sample. Next, we combined the positive and negative samples, and split all the samples into 7:3 randomly, 10-fold cross validation was used in every training process.

### Downstream analysis

To facilitate the further interpretation of predicted loops, the web server integrated two commonly used downstream analysis tools. Firstly, motif analysis accompanying the gene expression level was used to identify the transcription factors bounded near the loop anchor regions, the significantly enriched motifs were shown with a user-interactive table as well as bubble plot, which was implemented by Apache Echart (31). And the motif analysis was implemented by HOMER package (32) (Supplementary Figure S3). Secondly, GO enrichment analysis was used to annotate the loop anchors, the significantly enriched terms could be selected and shown in bar blot. The package of clusterProfiler (33) was integrated to perform the GO enrichment analysis (Supplementary Figure S4). The running time for motif analysis in the example case (>500k input file) was 15 ± 3 seconds, and the running time for GO analysis was 10 ± 3 seconds. For the input file larger than 500k and less than 1M, the running time of motif analysis and GO analysis ranged from 50 seconds to 1min.

### Visualization implementation

The web server provided visualization for the predicted E-P interactions, as well as the published loops identified from Hi-C, ChIA-PET, HiChIP and other 3C-based techniques. The visualization module integrated Ideogram API, which supported viewing the distribution of loops in a genome-wide scale, and supported the annotation of loops with regulatory elements and risk SNPs. Specific chromatin could be selected from the web page and zoomed in to check the interactions between genes and enhancers (Supplementary Figure S5). The visualization results could be exported into high-quality images and available for download.

### Input format description

Multi-omics datasets were required for generating features, which can be uploaded as tab-separated text files. The input multi-omics files can be ChIP-seq peaks of interested histone marks or transcription factor, RNA-seq expression profile, ATAC-seq peaks, etc. The peak files should be standard Broadpeak or Narrowpeak format of ENCODE (3) with at least 6 columns, the expression profile should be 2 columns with gene name and corresponding expression value. According to the testing of different input amount, the more input files, the more beneficial to the training process. And the web server achieved a near optimal performance with three input files, each file can be used to generate multiple features for different regions.

Chromatin loops was an optional input in BEDPE format, which can be generated by 3C-based techniques, with at least 6 columns containing the chromosome of loops and the coordinates of two anchors, if there were loop counts available, the counting values should be the 7^th^ column. For the ChIA-PET and HiChIP data, the analysis results could be directly transformed to BEDPE format and used as input. For the Hi-C data, user needs to call loops using adequate tools, such as Mustache (34), HiCExplorer (35), etc.

### Output format description

The EPIXplorer generated predicted results as well as high-quality images for download. For the supervised methods, the predicted results included: the generated features in plain text, the binary trained model file, the predicted E-P interactions which was annotated by enhancers and promoters, and the visual file for next step. For the unsupervised methods, the predicted result was only E-P interactions.

The generated features and binary trained model file were provided for users to re-implement the prediction procedure, the binary trained model was packed by scikit-learn (36), through which users could construct their own machine learning model conveniently. The predicted E-P interaction was a tab-separated text file with three columns, the first two columns were annotation of regulatory elements for two anchors, the third column was the confident score of the corresponding loop, the higher the score, the more reliable the loop.

In the motif analysis function of Downstream module, all the detected motifs and corresponding RNA-seq expression values were showed in an interactive table, users could select what they interested to present in the bubble plot. The size of each bubble indicated the enrichment level of motif, then the order of bubble was determined by normalized gene expression value. In GO analysis, all the GO terms, KEGG terms and Reactome terms were listed in an interactive table, and to be selected to generate a bar plot. The identities of terms were listed on the left side of bar plot, and the color of bars indicated the adjusted p-value of enrichment. Both bubble plot and bar plot with high-quality could be downloaded from the web page.

## CONCLUSION AND DISCUSSION

EPIXplorer allows to investigate E-P interactions from a variety of epigenomic datasets by integrating 9 robust predictive algorithms. The server supports different types of input to satisfy users’ requirements, the output from EPIXplorer is scored, fully annotated by regulatory elements and risk single-nucleotide polymorphisms (SNPs). Downstream analysis (motif analysis and GO enrichment) and Visualization benefits the non-computational biologists to explore the biological function of E-P interactions. Overall, EPIXplorer provides a user-friendly platform to predict the E-P interactions and explore the functional complexity of 3D genome. The web server makes it possible to study the pathological mechanism of GWAS SNPs under the 3D genome architecture.

EPIXplorer still faces some limitations, which need to be improved in the future. Firstly, TAD is a fundamental unit of the chromosomal structure, and greatly limits the formation of regulatory interactions between different domains (37). Although the E-P interactions usually locate inside the TAD structure, recent study has revealed that E-P interactions can cross TAD boundaries, and these boundary-crossing interactions largely correlate with transcriptional output (38). Therefore, it is necessary to learn about the relationships between predicted E-P interactions and TADs, which helps understand the formation of loops, as well as investigate the regulatory function of predicted loops. From this point of view, the predicted loops can be viewed cooperated with the Hi-C matrix and epigenetic data in the specific cell type. And the visualization results could be exported as universal formats, such as cool or hic, which can be transformed to the other tools to produce publication quality plots, like HiGlass (39). Secondly, the server can only predict the E-P interactions for human genome, which should consider in the future a possibility to work for a wider species and bio-samples. Cross-species/cross-cell lines prediction is an increasing demand for non-computational biologists.

## DATA AVAILABILITY

The datasets used for case study are available in the NCBI repository: K562-H3K27ac ChIA-PET: GSE59395; K562-H3K27ac HiChIP: GSE101498; K562 H3K27ac/ H3K4me1/ H3K4me3/ H3K27me3 ChIP-seq: GSE51334; K562 CTCF ChIP-seq: GSE51334 K562 ATAC-seq: GSE99173.

## Supplementary Material

gkac397_Supplemental_File

## References

[1] Prescott S.L. , Srinivasan R., Marchetto M.C., Grishina I., Narvaiza I., Selleri L., Gage F.H., Swigut T., Wysocka J. Enhancer divergence and cis-regulatory evolution in the human and chimp neural crest. Cell. 2015; 163:68–83. 26365491 10.1016/j.cell.2015.08.036 PMC4848043

[2] Levine M. Transcriptional enhancers in animal development and evolution. Curr Biol. 2010; 20:R754–R763. 20833320 10.1016/j.cub.2010.06.070 PMC4280268

[3] The ENCODE Project Consortium An integrated encyclopedia of DNA elements in the human genome. Nature. 2012; 489:57. 22955616 10.1038/nature11247 PMC3439153

[4] Zinzen R.P. , Girardot C., Gagneur J., Braun M., Furlong E.E.M. Combinatorial binding predicts spatio-temporal cis-regulatory activity. Nature. 2009; 462:65. 19890324 10.1038/nature08531

[5] Pennacchio L.A. , Ahituv N., Moses A.M., Prabhakar S., Nobrega M.A., Shoukry M., Minovitsky S., Dubchak I., Holt A., Lewis K.D. et al. In vivo enhancer analysis of human conserved non-coding sequences. Nature. 2006; 444:499. 17086198 10.1038/nature05295

[6] Dekker J. , Rippe K., Dekker M., Kleckner N. Capturing chromosome conformation. Science. 2002; 295:1306–1311. 11847345 10.1126/science.1067799

[7] Mifsud B. , Tavares-Cadete F., Young A.N., Sugar R., Schoenfelder S., Ferreira L., Wingett S.W., Andrews S., Grey W., Ewels P.A. et al. Mapping long-range promoter contacts in human cells with high-resolution capture Hi-C. Nature Genetics. 2015; 47:598–606. 25938943 10.1038/ng.3286

[8] Fullwood M.J. , Liu M.H., Pan Y.F., Liu J., Xu H., Mohamed Y.B., Orlov Y.L., Velkov S., Ho A., Mei P.H. et al. An oestrogen-receptor-α-bound human chromatin interactome. Nature. 2009; 462:58. 19890323 10.1038/nature08497 PMC2774924

[9] Mumbach M.R. , Rubin A.J., Flynn R.A., Dai C., Khavari P.A., Greenleaf W.J., Chang H.Y. HiChIP: efficient and sensitive analysis of protein-directed genome architecture. Nat Methods. 2016; 13:919–922. 27643841 10.1038/nmeth.3999 PMC5501173

[10] Mora A. , Sandve G.K., Gabrielsen O.S., Eskeland R. In the loop: promoter–enhancer interactions and bioinformatics. Brief Bioinform. 2016; 17:980–995. 26586731 10.1093/bib/bbv097 PMC5142009

[11] Xu H. , Zhang S., Yi X., Plewczynski D., Li M.J. Exploring 3D chromatin contacts in gene regulation: the evolution of approaches for the identification of functional enhancer-promoter interaction. Comput Struct Biotechnology J. 2020; 18:558–570. 10.1016/j.csbj.2020.02.013 PMC7090358 32226593

[12] Corradin O. , Saiakhova A., Akhtar-Zaidi B., Myeroff L., Willis J., Cowper-Sal·lari R., Lupien M., Markowitz S., Scacheri P.C. Combinatorial effects of multiple enhancer variants in linkage disequilibrium dictate levels of gene expression to confer susceptibility to common traits. Genome Res. 2014; 24:1–13. 24196873 10.1101/gr.164079.113 PMC3875850

[13] Naville M. , Ishibashi M., Ferg M., Bengani H., Rinkwitz S., Krecsmarik M., Hawkins T.A., Wilson S.W., Manning E., Chilamakuri C.S.R. et al. Long-range evolutionary constraints reveal cis-regulatory interactions on the human x chromosome. Nat Commun. 2015; 6:6904. 25908307 10.1038/ncomms7904 PMC4423230

[14] Ernst J. , Kheradpour P., Mikkelsen T.S., Shoresh N., Ward L.D., Epstein C.B., Zhang X., Wang L., Issner R., Coyne M. et al. Mapping and analysis of chromatin state dynamics in nine human cell types. Nature. 2011; 473:43–49. 21441907 10.1038/nature09906 PMC3088773

[15] Thurman R.E. , Rynes E., Humbert R., Vierstra J., Maurano M.T., Haugen E., Sheffield N.C., Stergachis A.B., Wang H., Vernot B. et al. The accessible chromatin landscape of the human genome. Nature. 2012; 489:75–82. 22955617 10.1038/nature11232 PMC3721348

[16] Zhu Y. , Chen Z., Zhang K., Wang M., Medovoy D., Whitaker J.W., Ding B., Li N., Zheng L., Wang W. Constructing 3D interaction maps from 1D epigenomes. Nat Commun. 2016; 7:10812. 26960733 10.1038/ncomms10812 PMC4792925

[17] Roy S. , Siahpirani A.F., Chasman D., Knaack S., Ay F., Stewart R., Wilson M., Sridharan R. A predictive modeling approach for cell line-specific long-range regulatory interactions. Nucleic Acids Res. 2015; 43:8694–8712. 26338778 10.1093/nar/gkv865 PMC4605315

[18] Dzida T. , Iqbal M., Charapitsa I., Reid G., Stunnenberg H., Matarese F., Grote K., Honkela A., Rattray M. Predicting stimulation-dependent enhancer-promoter interactions from chip-Seq time course data. Peerj. 2017; 5:e3742. 28970965 10.7717/peerj.3742 PMC5623311

[19] He B. , Chen C., Teng L., Tan K. Global view of enhancer–promoter interactome in human cells. Proc National Acad Sci. 2014; 111:E2191–E2199. 10.1073/pnas.1320308111 PMC4040567 24821768

[20] Cao Q. , Anyansi C., Hu X., Xu L., Xiong L., Tang W., Mok M.T.S., Cheng C., Fan X., Gerstein M. et al. Reconstruction of enhancer-target networks in 935 samples of human primary cells, tissues and cell lines. Nat Genet. 2017; 49:1428–1436. 28869592 10.1038/ng.3950

[21] Whalen S. , Truty R.M., Pollard K.S. Enhancer–promoter interactions are encoded by complex genomic signatures on looping chromatin. Nat Genet. 2016; 48:ng.3539. 10.1038/ng.3539 PMC4910881 27064255

[22] Belokopytova P.S. , Nuriddinov M.A., Mozheiko E.A., Fishman D., Fishman V. Quantitative prediction of enhancer–promoter interactions. Genome Research. 2019; 30:72–84. 31804952 10.1101/gr.249367.119 PMC6961579

[23] Tang L. , Hill M.C., Wang J., Wang J., Martin J.F., Li M. Predicting unrecognized enhancer-mediated genome topology by an ensemble machine learning model. Genome Res. 2020; 30:1835–1845. 33184104 10.1101/gr.264606.120 PMC7706734

[24] Cao F. , Fullwood M.J. Inflated performance measures in enhancer–promoter interaction-prediction methods. Nat Genet. 2019; 51:1196–1198. 31332378 10.1038/s41588-019-0434-7

[25] Singh S. , Yang Y., Póczos B., Ma J. Predicting enhancer-promoter interaction from genomic sequence with deep neural networks. Quantitative Biology. 2019; 7:122–137. 34113473 10.1007/s40484-019-0154-0 PMC8188889

[26] Moore J.E. , Pratt H.E., Purcaro M.J., Weng Z. A curated benchmark of enhancer-gene interactions for evaluating enhancer-target gene prediction methods. Genome Biol. 2020; 21:17. 31969180 10.1186/s13059-019-1924-8 PMC6977301

[27] Tang L. , Hill M.C., Ellinor P.T., Li M. Bacon: a comprehensive computational benchmarking framework for evaluating targeted chromatin conformation capture-specific methodologies. Genome Biol. 2022; 23:30. 35063001 10.1186/s13059-021-02597-4 PMC8780810

[28] Fulco C.P. , Munschauer M., Anyoha R., Munson G., Grossman S.R., Perez E.M., Kane M., Cleary B., Lander E.S., Engreitz J.M. Systematic mapping of functional enhancer–promoter connections with CRISPR interference. Science. 2016; 354:769–773. 27708057 10.1126/science.aag2445 PMC5438575

[29] Frankish A. , Diekhans M., Ferreira A.-M., Johnson R., Jungreis I., Loveland J., Mudge J.M., Sisu C., Wright J., Armstrong J. et al. GENCODE reference annotation for the human and mouse genomes. Nucleic Acids Res. 2018; 47:D766–D773. 10.1093/nar/gky955 PMC6323946 30357393

[30] Quinlan A.R. , Hall I.M. BEDTools: a flexible suite of utilities for comparing genomic features. Bioinformatics. 2010; 26:841–842. 20110278 10.1093/bioinformatics/btq033 PMC2832824

[31] Li D. , Mei H., Shen Y., Su S., Zhang W., Wang J., Zu M., Chen W. ECharts: a declarative framework for rapid construction of web-based visualization. Vis Informatics. 2018; 2:136–146.

[32] Heinz S. , Benner C., Spann N., Bertolino E., Lin Y.C., Laslo P., Cheng J.X., Murre C., Singh H., Glass C.K. Simple combinations of lineage-determining transcription factors prime cis-Regulatory elements required for macrophage and b cell identities. Mol Cell. 2010; 38:576–589. 20513432 10.1016/j.molcel.2010.05.004 PMC2898526

[33] Yu G. , Wang L.-G., Han Y., He Q.-Y. clusterProfiler: an r package for comparing biological themes among gene clusters. Omics J Integr Biology. 2012; 16:284–287. 10.1089/omi.2011.0118 PMC3339379 22455463

[34] Ardakany A.R. , Gezer H.T., Lonardi S., Ay F. Mustache: multi-scale detection of chromatin loops from Hi-C and Micro-C maps using scale-space representation. Genome Biol. 2020; 21:256. 32998764 10.1186/s13059-020-02167-0 PMC7528378

[35] Wolff J. , Rabbani L., Gilsbach R., Richard G., Manke T., Backofen R., Grüning B.A. Galaxy hicexplorer 3: a web server for reproducible Hi-C, capture Hi-C and single-cell Hi-C data analysis, quality control and visualization. Nucleic Acids Res. 2020; 48:W177–W184. 32301980 10.1093/nar/gkaa220 PMC7319437

[36] Pedregosa F. , Varoquaux G., Gramfort A., Michel V., Thirion B., Grisel O., Blondel M., Müller A., Nothman J., Louppe G. et al. Scikit-learn: machine learning in python. 2012;

[37] Dixon J.R. , Selvaraj S., Yue F., Kim A., Li Y., Shen Y., Hu M., Liu J.S., Ren B. Topological domains in mammalian genomes identified by analysis of chromatin interactions. Nature. 2012; 485:376. 22495300 10.1038/nature11082 PMC3356448

[38] Hsieh T.-H.S. , Cattoglio C., Slobodyanyuk E., Hansen A.S., Darzacq X., Tjian R. Enhancer-promoter interactions and transcription are maintained upon acute loss of CTCF, cohesin, WAPL, and YY1. 2021; bioRxiv doi:14 July 2021, preprint: not peer reviewed 10.1101/2021.07.14.452365. PMC9729117 36471071

[39] Kerpedjiev P. , Abdennur N., Lekschas F., McCallum C., Dinkla K., Strobelt H., Luber J.M., Ouellette S.B., Azhir A., Kumar N. et al. HiGlass: web-based visual exploration and analysis of genome interaction maps. Genome Biol. 2018; 19:125. 30143029 10.1186/s13059-018-1486-1 PMC6109259

