# Supplementary material for: EPIXplorer: A web server for prediction, analysis and visualization of enhancer-promoter interactions
Source: Nucleic Acids Res. 2022 May 25;50(W1):W290–7. doi: 10.1093/nar/gkac397 (PMC9252822; doi:10.1093/nar/gkac397)
Supplement: gkac397_Supplemental_File [file gkac397_supplemental_file.pdf]

Supplemental information for

EPIXplorer: A web server for prediction, analysis and visualization of enhancer-promoter interactions

Li Tang[1], Zhizhou Zhong[1], Yisheng Lin[1], Yifei Yang[1], Jun Wang[2], James F. Martin[3,4,5], Min Li[1*]

[1]Hunan Provincial Key Lab on Bioinformatics, School of Computer Science and Engineering, Central South University, Changsha 410083, China

[2]Department of Pediatrics, McGovern Medical School, The University of Texas Health Science Center at Houston, Houston, TX 77030, USA

[3]Department of Molecular Physiology and Biophysics, Baylor College of Medicine, Houston, TX 77030, USA

[4]Cardiovascular Research Institute, Baylor College of Medicine, Houston, TX 77030, USA

[5]Texas Heart Institute, Houston, TX 77030, USA

* To whom correspondence should be addressed. Tel: +86-0731-88879560; Email: limin@mail.csu.edu.cn

Table S1. BENGI datasets used for AUPR performance evaluation

	Data type	Reference	Positive	Negative
K562 BENGI	Hi-C loops	Rao *et al.* (2014) *Cell*	1,920	7,680
	crisprQTLs	Gasperini et al. (2019) *Cell*	589	2,356
GM12878 BENGI	RNAPII ChIA-PET	Tang et al. (2015) Cell	10,441	41,764
	CTCF ChIA-PET	Tang et al. (2015) Cell	3,794	15,176
	Hi-C loops	Rao *et al.* (2014) *Cell*	2,091	8,364
	CHi-C	Mifsud *et al.* (2015) *Nature Genetics*	19,519	78,076

Table S2. Gold standard loop sets used for ACC performance evaluation

	Antibody	Source	Positive	Negative
K562 Gold loop	POLR2A	CRISPRi contacts (Fuentes et al. 2018, Fulco et al. 2016, Fulco et al. 2019, Gasperini et al. 2019, Huang et al. 2018, Klann et al. 2017, Liu et al. 2017, Mumbach et al. 2017, Nasser et al. 2021, Qi et al. 2018, Spisak et al. 2015, Tewhey et al. 2016, Thakore et al. 2015, Ulirsch et al. 2016, Wakabayashi et al. 2016, Wang et al. 2017, Xie et al. 2017, Xu et al. 2015), eQTL (GEUVADIS, GTEx) ENCODE high confident loops	9,994	9,994
	CTCF		433,783	433,783
	H3K27ac		1,569	1,569
	H3K4me1		3,627	3,627
	H3K4me2		658	658
	H3K4me3		1,368	1,368
	YY1		459,590	459,590
GM12878 Gold loop	RAD21		20,051	20,051

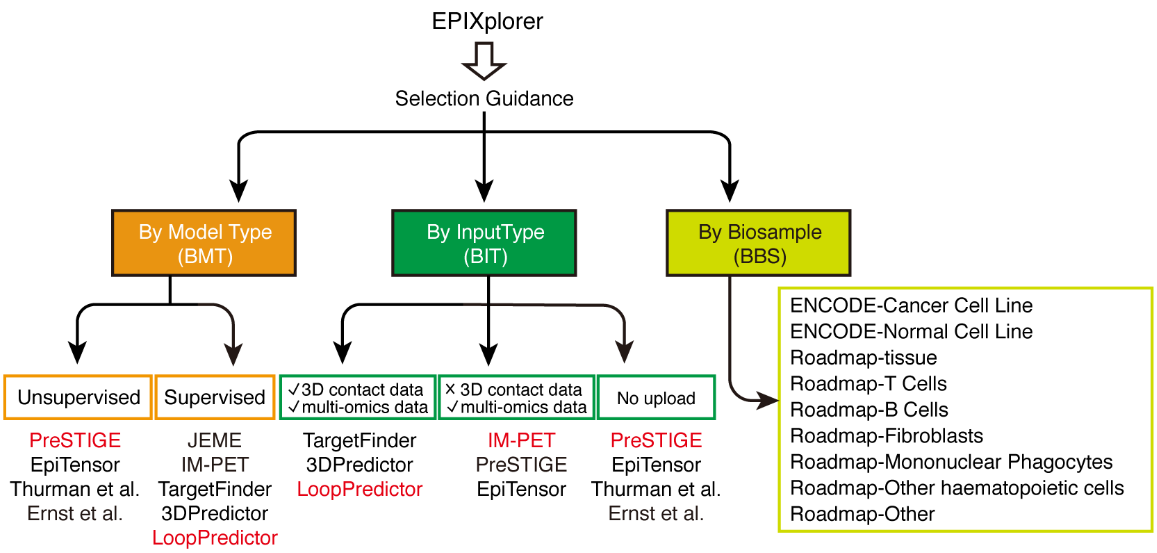

Figure S1. Selection Guidance and the corresponding algorithms for each category. BMT divides 9 algorithms into supervised and unsupervised according to the type of prediction model. BIT characterizes these algorithms by the input they support. BBS classifies all the supported cell lines into 9 major types and lists the available algorithms for each cell line. The best algorithms for each category are marked with red color.

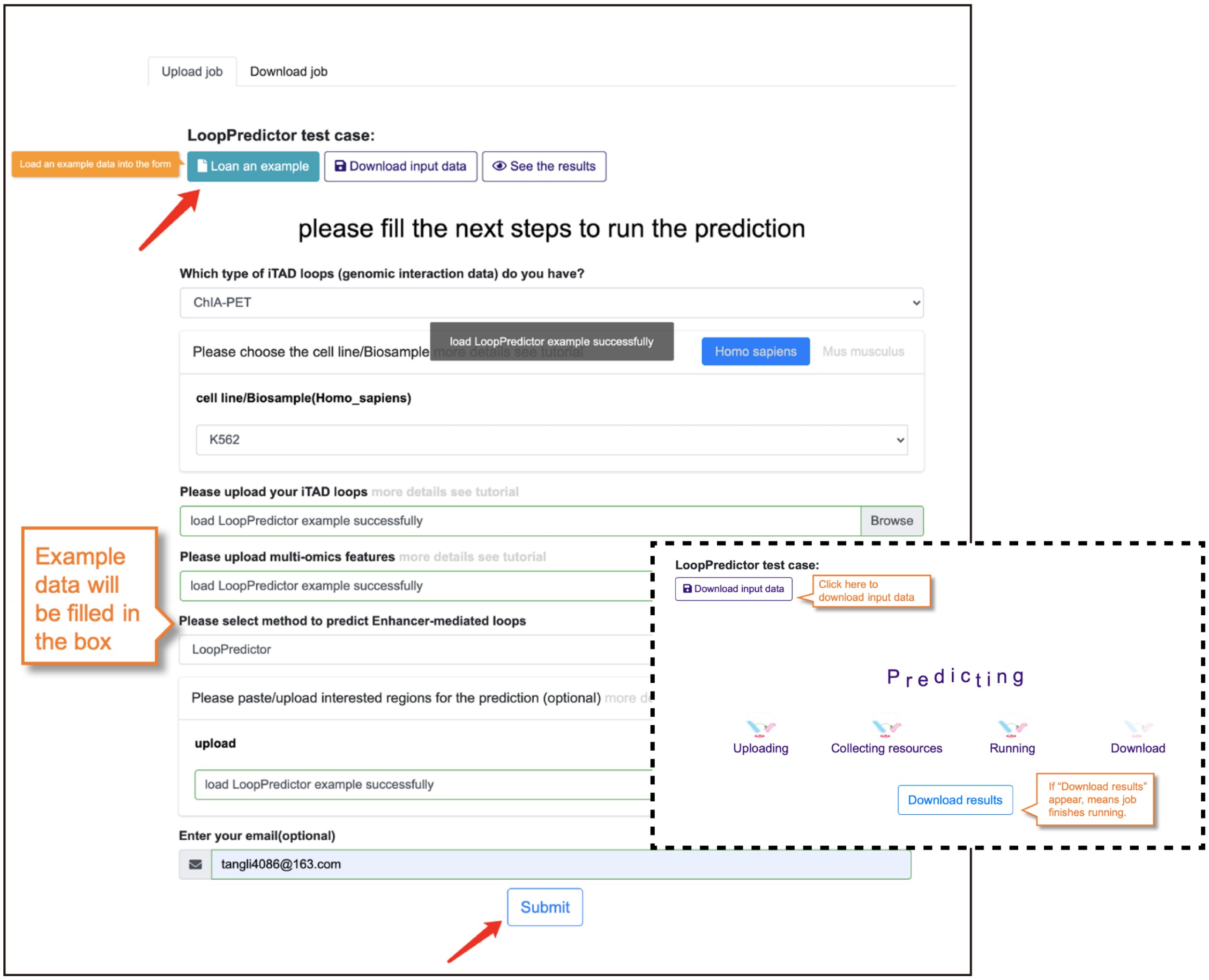

Figure S2. Load case of K562 datasets with LoopPredictor. The web server provides example case for each algorithm, here we use LoopPredictor as an example. Users can load the example data to input boxes by clicking “Load an example” button, then click “Submit” button to start the running task, the results can be downloaded when the task finish. To facilitate the interpretation of running procedure, the example input data is also available for download.

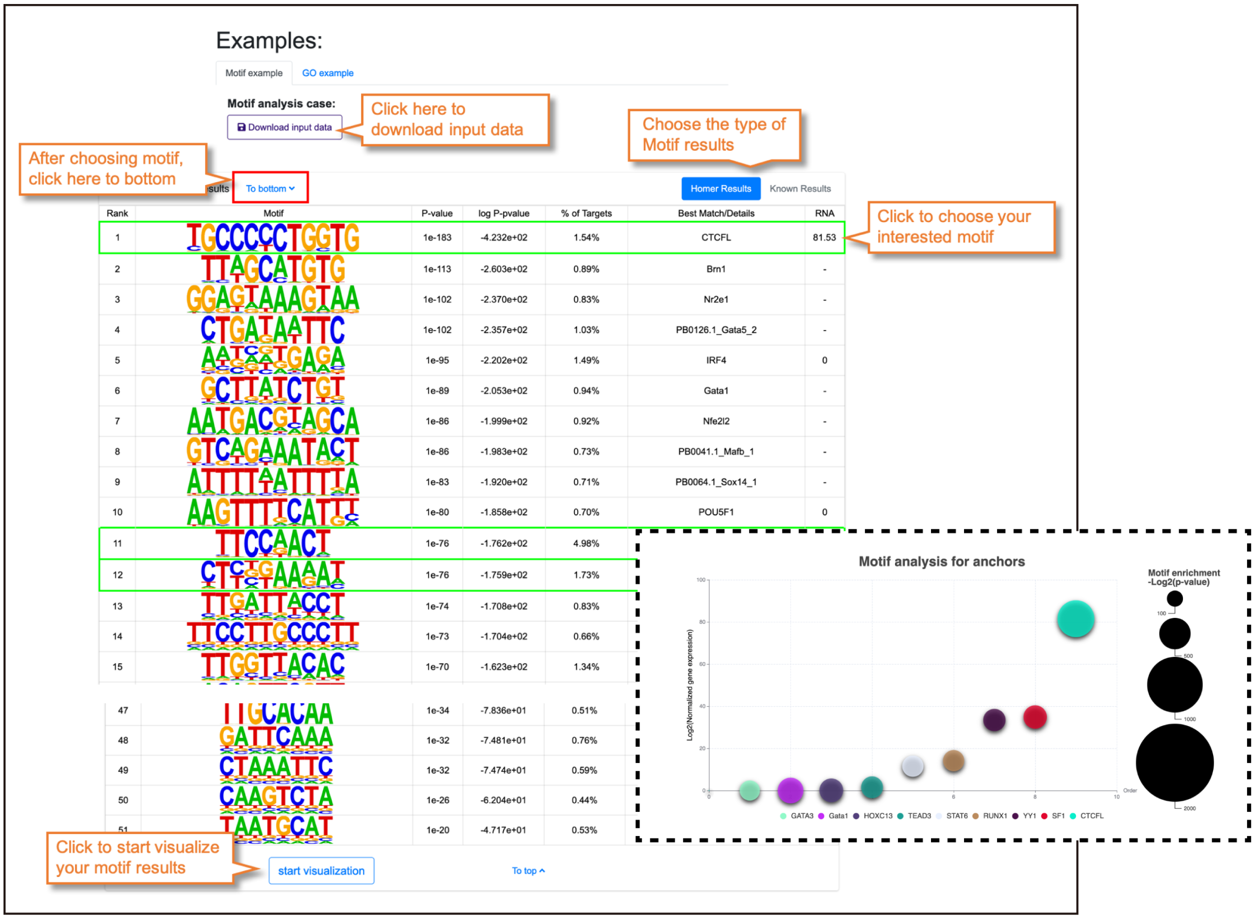

Figure S3. Motif analysis in Downstream module. The motif analysis is implemented by HOMER package accompanying RNA expression data, users can paste the job-id to fetch predicted loops, which can be transferred to the downstream module conveniently. The motif identification results are shown in an interactive table, users can select the interested motifs by clicking the corresponding table row. Then click “Start visualization” button to visualize the ranking of selected motifs through a bubble plot, and the high-quality plot can be downloaded.

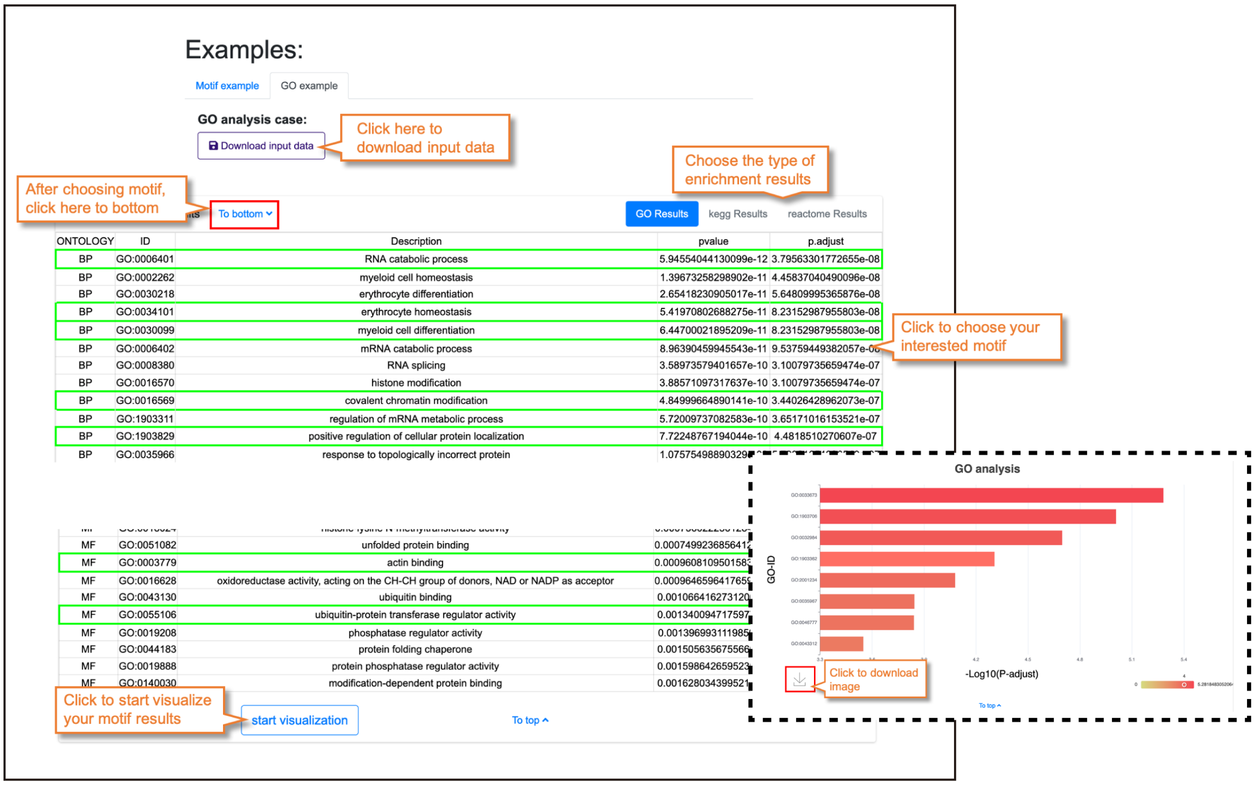

Figure S4. GO analysis in Downstream module. The GO analysis is implemented by clusterProfiler package. Similar as motif analysis, users can fetch and transfer their predicted results to this module with job-id. The enriched GO terms and corresponding p-values are shown in an interactive table, users can select the interested terms by clicking the table rows. The bar plot of GO terms will be generated when clicking “Start visualization”, and the high-quality plot is available to be downloaded.

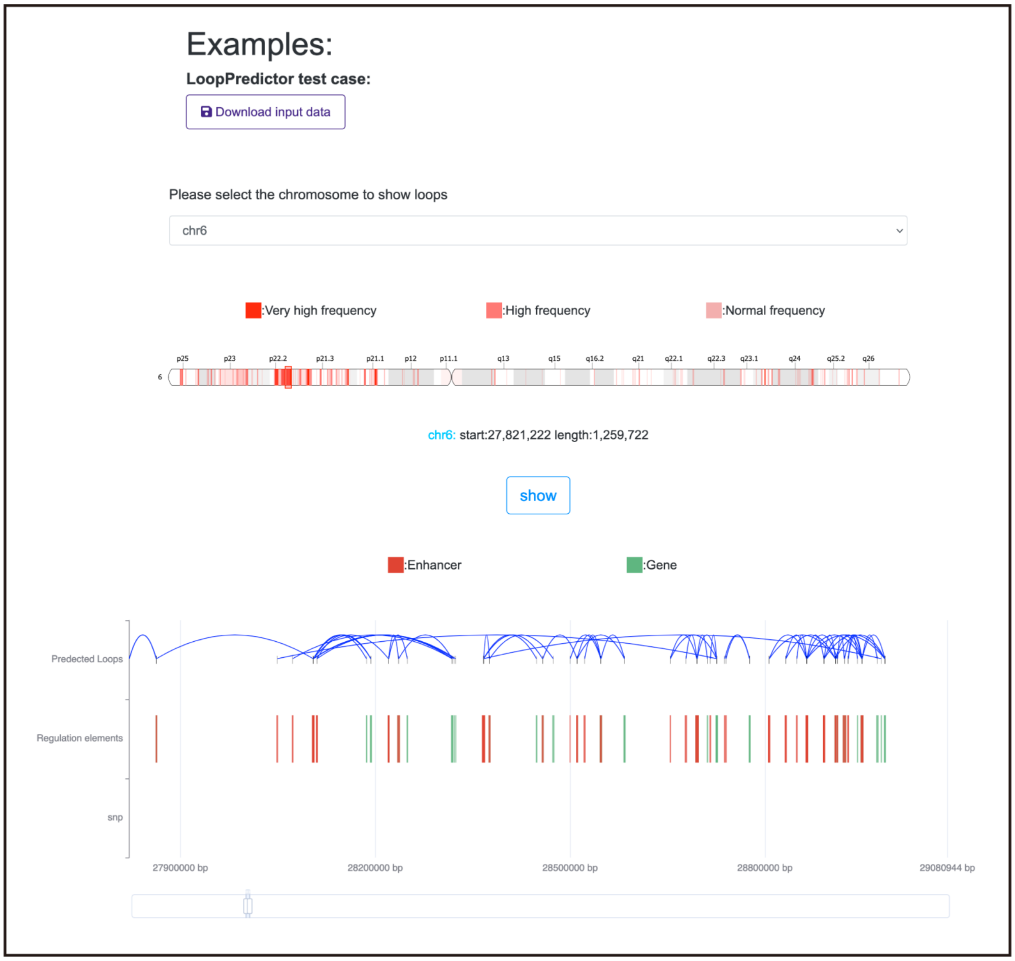

Figure S5. Visualization module. Similar as motif analysis and GO analysis, users can fetch and transfer their predicted results to this module with job-id. Users can select a specific chromosome to visualize, and the visualization can be zoomed in by dragging the chromosome. The loops are shown as arcs. To investigate the predicted results from genome-wide scale, the distribution of loops is also shown in the webpage.
